# Evaluation of temperature increase during in-office bleaching

**DOI:** 10.1590/1678-775720150154

**Published:** 2016

**Authors:** Rafael Francisco Lia MONDELLI, Ana Flávia SOARES, Eugenio Gabriel Kegler PANGRAZIO, Linda WANG, Sergio Kiyoshi ISHIKIRIAMA, Juliana Fraga Soares BOMBONATTI

**Affiliations:** Universidade de São Paulo, Faculdade de Odontologia de Bauru, Departamento de Dentística, Endodontia e Materiais Odontológicos, Bauru, SP, Brasil,

**Keywords:** Tooth bleaching, Light, Temperature

## Abstract

The use of light sources in the bleaching process reduces the time required and promotes satisfactory results. However, these light sources can cause an increase in the pulp temperature.

**Objective:**

The purpose of the present study was to measure the increase in intrapulpal temperature induced by different light-activated bleaching procedures with and without the use of a bleaching gel.

**Material and Methods:**

A human maxillary central incisor was sectioned 2 mm below the cementoenamel junction. A K-type thermocouple probe was introduced into the pulp chamber. A 35% hydrogen peroxide bleaching gel was applied to the vestibular tooth surface. The light units used were a conventional halogen, a hybrid light (only LED and LED/Laser), a high intensity LED, and a green LED light. Temperature increase values were compared by two-way ANOVA and Tukey´s tests (p<0.05).

**Results:**

There were statistically significant differences in temperature increases between the different light sources used and between the same light sources with and without the use of a bleaching gel. The presence of a bleaching gel generated an increase in intra-pulpal temperature in groups activated with halogen light, hybrid light, and high intensity LED. Compared to the other light sources, the conventional halogen lamp applied over the bleaching gel induced a signiﬁcant increase in temperature (3.83±0.41°C). The green LED unit with and without gel application did not produce any significant intrapulpal temperature variations.

**Conclusion:**

In the present study, the conventional halogen lamp caused the highest increase in intrapulpal temperature, and the green LED caused the least. There was an increase in temperature with all lights tested and the maximum temperature remained below the critical level (5.5°C). The addition of a bleaching gel led to a higher increase in intrapulpal temperatures.

## INTRODUCTION

Whitening process has been reinforced by the development of new technologies allowing patients to get faster and better results^16^ ^,^ ^22^. This technique can be performed at the office, at home, or a combination of both. In-office bleaching is usually performed using bleaching agents with high concentrations of hydrogen peroxide (15-38%). An external source of hybrid light (LED/Diode Laser) can be applied to activate the bleaching gel agent in less time with good results, with a small increase in the intrapulpal temperature, and less sensitivity^21^.

These external light sources have the ability to heat the hydrogen peroxide gel, thus increasing the rate of oxygen decomposition, which promotes bleaching^6^ ^,^ ^9^.

Although the use of hybrid light sources in the bleaching process reduces the time required for performing the process and promotes satisfactory results^21^, some of these light sources can cause an increase in the intrapulpal temperature, which can increase the incidence of post-operative sensitivity (55%-100%)^14^ ^,^ ^20^.

The equipment on the market includes: incandescent light sources, photopolymerizers, infrared lasers, and high power LEDs, among others. Among the benefits obtained by photo activation, only a small fraction of light is absorbed by the bleaching gel, and its energy is converted into heat^17^ ^,^ ^19^. The light, however, can accelerate the degradation of hydrogen peroxide with less time required for bleaching and increase the temperature of the bleaching agent and the dental pulp^21^.

Many different types of lights are currently used in dentistry for bleaching, including halogen, plasma arc, light emitting diodes (LED), diode lasers, carbon dioxide lasers (CO_2_), argon lasers, and non-thermal atmospheric pressure plasmas (NAPP). Most of the lights emitted by these lamps are within the visible spectrum, but some lamps also emit the light near an infrared or an ultraviolet spectrum. Some systems are designed solely to be used for tooth whitening, while others have several additional functions (bleaching and polymerization of restorative materials). When these lamps are used, an increase in gel temperature and intrapulpal temperature can be observed.

External heat applied to the teeth can cause pulp damage in varying degrees, depending on the magnitude and duration of the increase in temperature^17^. In an *in vivo* study with monkeys, an increase in temperature inside of the pulp chamber of 5.5°C caused irreversible pulpits^29^. Temperatures in the pulp chamber above 46°C can cause irreversible changes, such as stasis and thrombosis in the blood vessels^25^. Some authors claim that the highest temperature tolerated by human pulp^2^, 5.5°C, is considered the critical point of tolerance for intrapulpal temperature.

Mainly due to aggressive marketing of some companies, whitening is becoming increasingly popular and many products that use different energy sources and protocols are available on the market. However, they should be tested in such a way that safety parameters are maintained.

The aim of the present *in vitro* study was to evaluate the intrapulpal temperature increases generated by a selection of lights used as part of the bleaching process, alone or in combination with a bleaching gel.

## MATERIAL AND METHODS

This is an *in vitro* study, and the experimental design for analysis of temperature variation was divided into two study factors: light sources (in five levels: a green LED light, a high intensity LED light, a LED/Laser hybrid light, tested with and without laser activation, and a conventional halogen lamp) and treatment (in two levels: with or without bleaching gel), the design determined ten experimental groups (G1-G10) as described in Table 1.

**Table 1:** Mean values of temperature variation, standard deviation, and statistical analysis comparison between groups (Tukey’s test)

Groups	Light sources	Mean±SD
G1	D-Light Green	0.00±0^A^
G2	D-Light Green + gel	0.17±0.41^A^
G3	Smart Lite	2.17±0.41^C^
G4	Smart Lite + gel	3.00±0^D^
G5	UltraBlue IV	1.00±0^B^
G6	UltraBlue IV + gel	2.17±0.41^C^
G7	UltraBlue IV laser	1.17±0.41^B^
G8	UltraBlue IV laser + gel	2.33±0.52^CD^
G9	Spectrum	2.70±0.41^C^
G10	Spectrum + gel	3.83±0.41^E^

Different capital letters indicate statistically significant difference between lines (p<0.05)

### Material

**Figure 1:**
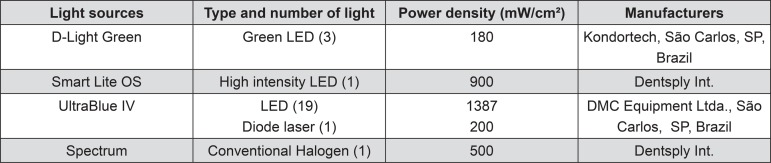
Light units studied

The manufacturer’s name and other information of the light sources used are listed in Figure 1. The bleaching agent is an in-office 35% hydrogen peroxide gel (Lase Peroxide, DMC Equipamentos Ltda., São Carlos, SP, Brazil). This product has a red color due to the addition of photoactive colorants (Urucum and Juá, Brazil). Other components include amide, glycol and water.

### Tooth preparation

**Figure 2:**
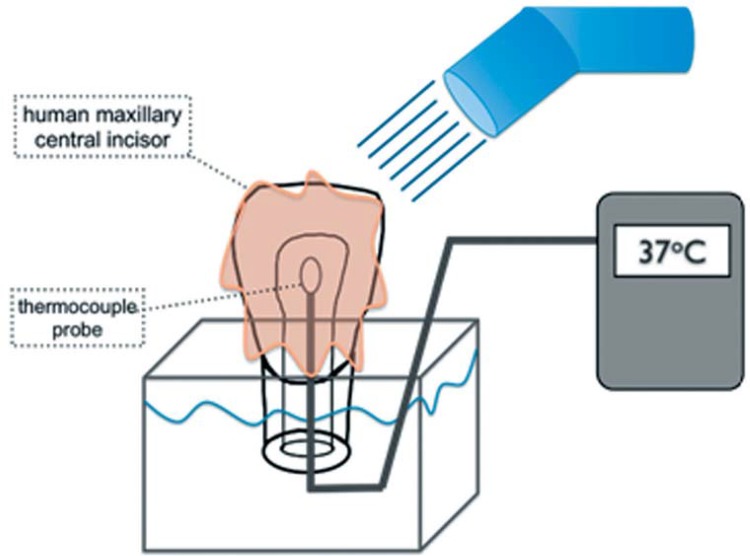
Schematic drawing of experimental set up showing the starting temperature before light activation

A single, human central upper incisor was used throughout the test. This tooth was donated from an organ bank of the Bauru School of Dentistry (FOB-USP), having obtained approval by the ethics committee of the institution through process no. 002/2008. The root of the tooth was sectioned 2 mm below the cementoenamel junction, perpendicular to the long axis of the tooth, using a slow speed diamond disc (Isomet, Buehler Ltd., Lake Bluff, IL, USA). The apical foramen of the root canal was enlarged using a #2135 diamond bur (KG Sorensen, Cotia, SP, Brazil) and all pulp tissue remnants were removed from the pulp chamber. The empty pulp chamber was filled with a heat absorption compound (Termal Paste, Implastec, Votorantim, SP, Brazil; 55.15.3243.3788) to simulate the pulp tissue as a heat conducting medium. A thin K-type thermocouple probe (MT 401A, Minilpa Ind. Com. Ltda., São Paulo, SP, Brazil) was inserted into the pulp chamber through the apical foramen and placed at its most coronal level. Its position was radiographically checked. During the testing procedure, the root surface of the tooth was partially submerged into a water bath at 37±1°C (Figure 2).

### Bleaching method

A 1.0 mm layer of 35% hydrogen peroxide-based bleaching gel was applied on the entire vestibular surface of the tooth. The distance between the emitting tip of the light source and the tooth surface was set at 10 mm. Light application protocols, as recommended by the manufacturer for each light source, were used. For the conventional halogen light and the high intensity LED units, 5 applications of 30 s each (with light) and 15 second intervals (without light) were utilized (this sequence is typically used in the clinic in order to avoid tooth overheating). For the hybrid LED/laser light, the protocol was 3 min of light activation and 1 min without light, followed by another 3 min of light activation. The green LED was applied for 1 min, followed by 5 min without light and then another 1 min with light activation.

### Temperature measurement

The room temperature was controlled at 24+1°C. Each of the light sources were applied with and without the bleaching gel on the tooth surface, for a total of 10 experimental groups. The gel was removed and a new layer was applied every time that a light source was applied to the tooth. At least 15 min were allowed in between the applications of all light sources in order to allow the temperature to return to baseline. Each measurement for all groups was repeated six times, and the temperature was recorded every 30 s during the test. The mean values of the differences between the initial and final temperature readings for each group was calculated.

This protocol was used based on the results of a pilot study performed prior to this study.

### Statistical analysis

Two-way analysis of variance (ANOVA) was used to determine if there were statistically significant differences between the groups (p≤0.05) and the Tukey´s test was used for all comparisons among the groups (p≤0.05). The program used for statistical analysis was the Statistica for Windows (version 6.0 StatSoft, Tulsa, Oklahoma, USA).

## RESULTS

There were statistically significant differences in the temperature increases among the different light sources and the same light sources, with and without the presence of a bleaching gel. The interaction between the light source and the gel was also significant.

**Figure 3:**
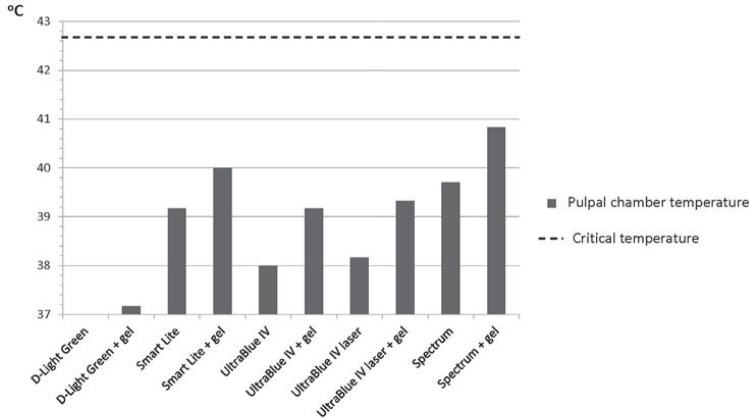
Intra-pulpal temperature increase during the in-office bleaching procedures for the different experimental groups

Compared to the other light sources, the conventional halogen lamp applied over the bleaching gel induced the greatest increase in temperature (3.83±0.41°C) (Table 1, Figure 3). The green LED light unit, with and without gel application, did not produce any significant temperature variation. The application of the bleaching gel significantly promoted the temperature increases in all the other units.

## DISCUSSION

Procedures with bleaching agents in high concentrations, coupled with a longer exposure time, can damage the pulp tissue^8^ ^,^ ^28^. In addition, the temperature variations of the intra-pulpal chamber has been a reason for concern^27^.

An increase in real temperature in the intra-pulpal chamber was observed for all groups evaluated, with the exception of the groups who used the green LED light source, possibly due to the low power density^10^. The higher temperature of the intra-pulpal chamber was achieved with the use of a conventional halogen lamp coupled with a bleaching gel, which corroborates previous studies^15^ ^,^ ^30^. This lamp was widely used at the onset of the in-office bleaching with the use of a light source, but its unstable temperature increases and, consequently, tooth sensitivity has caused it to fall into disuse.

The influence of the diode laser was minimal regarding the increase in temperature when the hybrid light source was also used, probably due to the low power density of the diode laser. This observation is significant because 1W, 2W, and 3W diode lasers are harmful to the pulp, but when used with a low power density, may have therapeutic qualities while reducing inflammatory reactions^13^. Although the present study did not evaluate the sensitivity, the clinical use of this therapeutic laser during bleaching may lead to a reduction in post-operative sensitivity^3^.

In order to increase the absorption of light, and consequently the temperature, thus optimizing this effect, some specific bleaching gel colorants are added to the bleaching agents such as red dye (Urucum and Juá, presented in Lase Peroxide, DMC Equipamentos Ltda., São Carlos, SP, Brazil). The groups which used the gel experienced a significantly higher increase in temperature, as shown in previous studies^5^ ^,^ ^11^. In contrast, the application of a bleaching gel or vaseline has been shown to act as a physical barrier, causing a decrease in the intrapulpal chamber temperature^17^.

An increase of 5.5°C in the intrapulpal temperature has been shown to cause irreversible pulpitis in 15% of the monkey teeth tested^29^. However, extrapolation of values obtained from humans to monkeys is highly questionable^7^. In the present study, an increase of 5.5°C in the intra-pulpal temperature is regarded as the threshold, which should not increase to prevent irreversible damage to the pulp.

Some studies have measured the pulp temperature increases induced by the bleaching gel when the tooth is exposed to different energy sources. The CO_2_ lasers, diode lasers^30^, conventional halogen lamps, and LED^11^ with high densities reported temperature values above the critical temperature. However, another study reported a lower temperature increase with halogen lamps, Plasma arcs, LED, and LED/Lasers^11^. It is very difficult to compare the different studies and evaluate their results because of the number of variables present such as time, energy, initial temperature of the pulp chamber, and distance from the photoactivator tip^9^.

Although an attempt *in vitro* was made to simulate the temperature of the pulp chamber, these results should be interpreted with a certain amount of caution. The pulpal blood flow acts as a heat diffuser, with the pulp circulation capable of dissipating some heat prior to damaging the pulp cells^18^ ^,^ ^24^. The current *in vitro* model was unable to replicate the pulp flow, therefore, it is possible that the increase in temperature was lower. The heating of the bleaching agent not only leads to an increase in temperature in the intra-pulpal chamber, but also a greater penetration of peroxide in the pulp chamber^4^.

The diffusion of hydrogen peroxide in the pulp leads to oxidative stress, which could adversely affect the cell metabolism of the pulp^6^. Histological evaluations of extracted human teeth have shown that heat can cause mild inflammatory responses. In some cases, however, it could also be caused by the use of 35% H_2_O_2_^1^.

Clinically, patients treated with bleaching agents activated with heat often complain of dental hypersensitivity up to 48 h after the bleaching procedure^12^.

Light sources have been widely used to accelerate the whitening process, with advantages such as an immediate lower tooth sensitivity^23^ ^,^ ^26^ ^,^ ^28^, besides decreasing the clinical time^21^. However, more studies are needed to establish the precise correlations between the new light sources and bleaching gels available in the market and its implication to the pulp organ.

## CONCLUSION

Within the limitations of the present *in vitro* study, the results showed that the conventional halogen lamp caused the greatest increase in intra-pulpal temperature and the green LED the lowest.

There was an increase in intra-pulpal temperature with all light sources tested, and the maximum temperature remained below the critical level (5.5°C).The addition of bleaching gel led to a higher increase in intra-pulpal temperatures.
